# First Complete Sequence of a Giant Linear Plasmid from a *Micrococcus* Strain Isolated from an Extremely High-Altitude Lake

**DOI:** 10.1128/genomeA.00885-13

**Published:** 2013-11-27

**Authors:** Julián Rafael Dib, Jörg Schuldes, Andrea Thürmer, María E. Farias, Rolf Daniel, Friedhelm Meinhardt

**Affiliations:** PROIMI-CONICET, Tucumán, Argentinaa; Institut für Molekulare Mikrobiologie und Biotechnologie, Westfälische Wilhelms-Universität Münster, Münster, Germanyb; Genomische und Angewandte Mikrobiologie, Laboratorium für Genomanalyse, Institut für Mikrobiologie und Genetik, Georg-August-Universität Göttingen, Göttingen, Germanyc

## Abstract

*Micrococcus* sp. strain V7, an actinobacterial strain adapted to the extreme conditions of the Laguna Vilama, an extremely high-altitude (4,600 m above sea level) lake in the Argentinian Puna, was found to carry the giant linear plasmid pLMV7. We determined its sequence (92,815 bp) as a prerequisite to the investigation of its role in survival in such a harsh environment.

## GENOME ANNOUNCEMENT

High-altitude lakes in the Argentinian Puna are extreme and pristine environments, but organisms living in such habitats must adapt to very harsh conditions, such as high UV radiation, oligothrophy, and a high arsenic concentration. Among the isolated actinobacteria from Laguna Vilama (4,600 m above sea level), *Micrococcus* sp. strain V7 displays multiple resistances, i.e., to antibiotics, UV irradiation, and heavy metals (1, 2). DNA analysis by pulsed-field gel electrophoresis disclosed the presence of a 92-kb linear extrachromosomal genetic element, which has terminal proteins covalently attached to the 5′-ends; the linear plasmid was termed pLMV7 (3). As the host adaptation traits might be at least partially conferred by pLMV7, the plasmid was fully sequenced, being to the best of our knowledge the first accessory linear genetic element that has been completely sequenced in the genus *Micrococcus*.

Purified plasmid was sequenced by combining Sanger sequencing and 454 pyrosequencing. A plasmid library was constructed with the TOPO TA kit (Life Technologies, Darmstadt, Germany). In total, 576 recombinant plasmids were end sequenced with an ABI 3730xl automated DNA sequencer (Life Technologies, Darmstadt, Germany), processed with Phred, and assembled using Phrap (http://www.phrap.org). The 454 shotgun library was sequenced with the Genome Sequencer FLX system (454 Life Sciences, Roche Applied Science, Branford, CT) using titanium chemistry. About 86,445 shotgun reads were generated and assembled *de novo* into 9 large contigs (>500 bp) using the Roche Newbler assembler software 1.1 (454 Life Sciences, Roche Applied Science). Finally, the contigs generated by the Sanger sequencing approach and 454 were joined.

Sequence editing was done using GAP4 as part of the Staden software package (4), and final gap closure was performed by PCR and primer walking using the Bio-X-Act kit (Bioline, London, United Kingdom). Finally, the terminal inverted repeats (TIRs) were sequenced by two different approaches. The 3′-end of the plasmid was cut with NsiI, and the generated fragments were purified and inserted into a cloning vector according to the protocol of Hirochika et al. (5). The other end of the plasmid was sequenced by the restriction of the plasmid with SpeI, which cuts close the unsequenced telomeric terminus. Subsequently, the generated fragments were self-ligated, and PCR sequencing of the unknown DNA was performed (6).

The complete nucleotide sequence of pLMV7 (69.4% G+C) consists of 92,815 bp comprising 114 protein-coding genes, 28 (25%) of which were assigned to known functions.

A perfect TIR of 732 bp was found along with further but interrupted homologous sequences outside this region. Due to structural similarities, it is assumed that the TIRs are instrumental in the replication of the termini as for other similarly structured linear genomes (7).

Further analysis of the plasmid sequence will probably clarify the potential function of the plasmid for the survival or adaptation of its host to harsh environments. Furthermore, analysis of the TIRs may contribute to the understanding of the still-obscure mechanism involved in the replication of linear plasmids and linear chromosomes.

### Nucleotide sequence accession number.

The whole sequence of plasmid pLMV7 has been deposited in GenBank under the accession no. KF577591.
